# Physiology Based Approaches for Breeding of Next-Generation Food Legumes

**DOI:** 10.3390/plants7030072

**Published:** 2018-09-08

**Authors:** Arun S. K. Shunmugam, Udhaya Kannan, Yunfei Jiang, Ketema A. Daba, Linda Y. Gorim

**Affiliations:** 1 Department of Plant Science, University of Saskatchewan, 51 Campus Drive, Saskatoon, SK S7N5A8, Canada; udhaya.kannan@usask.ca (U.K.); kea530@usask.ca (K.A.D.); linda.gorim@usask.ca (L.Y.G.); 2 Agriculture and Agri-Food Canada, Saskatoon Research and Development Center, 107 Science Place, Saskatoon, SK S7N0X2, Canada; 3 Department of Plant Agriculture, University of Guelph, 50 Stone Road E., Guelph, ON N1G2W1, Canada; yjiang12@uoguelph.ca

**Keywords:** legume breeding, food security, physiology, abiotic stress, genomics, gene editing

## Abstract

Plant breeders and agricultural scientists of the 21st century are challenged to increase the yield potentials of crops to feed the growing world population. Climate change, the resultant stresses and increasing nutrient deficiencies are factors that are to be considered in designing modern plant breeding pipelines. Underutilized food legumes have the potential to address these issues and ensure food security in developing nations of the world. Food legumes in the past have drawn limited research funding and technological attention when compared to cereal crops. Physiological breeding strategies that were proven to be successful in cereals are to be adapted to legume crop improvement to realize their potential. The gap between breeders and physiologists should be narrowed by collaborative approaches to understand complex traits in legumes. This review discusses the potential of physiology based approaches in food legume breeding and how they impact yield gains and abiotic stress tolerance in these crops. The influence of roots and root system architectures in food legumes’ breeding is also discussed. Molecular breeding to map the relevant physiological traits and the potentials of gene editing those traits are detailed. It is imperative to unlock the potentials of these underutilized crops to attain sustainable environmental and nutritional food security.

## 1. Introduction

Feeding the nine billion people of the world in 2050 will only be possible by doubling the current agricultural crop production. This increase in crop production target has been heavily challenged by climate change and the environmental impact on agricultural systems [1]. As stated in Reference [2], adapting crop varieties and improving them to meet the global demand is an “endless task”. Plant breeding and agronomic improvements will have to adopt newer strategies to ensure food security. Breeders and the agricultural community around the world will have to expand the capacity of current breeding programs to increase yield potentials [3]. Yet another challenge to agricultural research is to address the problem of micronutrient deficiency, or “hidden hunger”, in developing countries. Though the green revolution resulted in significant poverty alleviation and hunger reduction, nutrition-related issues faced by poor parts of the world remain unsolved [4]. Around 815 million people in the world are malnourished due to the lack of essential mineral nutrients such as iron, zinc and manganese in their dietary habits [5]. Biofortification strategies are projected as a potential solution to tackle micronutrient deficiencies [6]. However, the diversity of crop species exploited for this purpose has been limited so far. In an urgent need for another green revolution, there should be a diversification of crop species that are to be included in breeding programs. “Orphan” or underutilized crops for examples finger millet, teff, yams, and various food legumes are rich in nutritional values, stress tolerant and have the potential to positively impact the health of people in developing countries [7]. Thus, the need of the hour is to design efficient strategies to breed diversified, climate-smart, nutrient efficient, and high yielding crop varieties.

Underutilized crops of the world can significantly contribute to global food security by reducing the reliance on major crops such as wheat, rice, maize, and soybean and fertilizers input. They also can increase the household income of farmers in poor nations, contribute to their balanced diet, and preserve their cultural food diversity [8]. Food legumes play several roles in this context. The consumption of food legumes has proven to increase the essential micronutrient intake in humans thereby reducing malnutrition. They are low in fat and sodium, rich in iron, potassium, protein, fibre and folate, cholesterol and gluten-free and have a low glycemic index [9]. They have also been linked to a reduction in weight, aging and stress, cancer and diabetes reduction and control [10]. Legumes through their biological nitrogen fixation, when included as inter-crops in cereal crops cultivation, have increased their yield that particular year and also in consecutive years [11]. They have the potential to regulate their biological nitrogen fixation depending on the availability of resources in a specific environment to maintain productivity [12]. Despite their vital roles in food security and sustainable agriculture, legumes gain lesser research and development attention when compared to major cereals. It is evident that this neglect on legumes had negatively affected the world’s nutritional and environmental sustainability [13]. Recognizing their potentials and to draw attention towards pulse crops improvement, the United Nations (UN) and Food and Agricultural Organization (FAO) have declared 2016 as the international year of pulses. A list of international and national programs and centers that focus on pulse crops improvement has been listed in Reference [14]. These organizations collectively work towards breeding better legume quality crops and socio-economic improvement of people that rely on them. In a changing world, attempting to increase yield stability, rather than just practicing a traditional empirical approach, plant breeders will also have to focus on the physiological aspects of crops to achieve their yield goals. Physiology-based phenotyping for traits of specific interests is significant in crop improvement programs of the 21st century.

**Figure 1:**
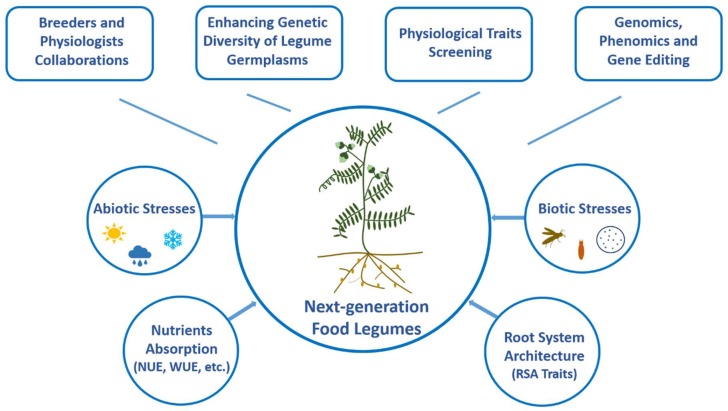
A conceptual framework to design next-generation food legumes. Climate-resilient, nutrient efficient, and high yielding pulse crops can be developed by collaborative efforts of breeders and physiologists leveraging the genomics, phenomics, and gene editing advancements.

An increase in yield depends on a myriad number of physiological traits including, but not limited to light, water and nutrient use efficiency of crops. Root architecture, its sink strength and translocation, water and nutrient acquisition and a canopy’s efficiency in capturing light, and photosynthesis efficiency are some of the factors which largely contribute to crop yield [15]. Drought stress adds a layer of complexity to phenotype physiological traits that are associated with yield gains. The importance of translating the beneficial traits that are screened during drought in controlled environmental conditions to field environments in order to be useful to breeders has been emphasized by physiologists [16]. These traits can then be used either directly in selection or used as surrogates to identify superior crop varieties. “Phenomics” or next generation phenotyping techniques will play a pivotal role in unblocking the bottlenecks in phenotyping physiological traits. By employing high throughput phenotyping strategies at the field and greenhouse level in individual steps of a breeding pipeline, breeders can overcome the limitations of physiological phenotyping. To narrow the genotype to phenotype information gap, there are newer technologies such as red, green and blue (RGB), laser, stereo, multispectral, thermal, hyperspectral, fluorescence imaging, magnetic resonance imaging (MRI) and computed tomography (CT) based screening that can be employed in field environments [17,18]. These technologies, coupled with physiological breeding and when used to screen new crop ideotypes generated from diversified genetic resources, will significantly improve crop genetic gains [19]. This approach has clearly benefited the CIMMYT wheat breeding program, which is evident from the release of a new and improved generation of wheat lines that showed increased genetic gains. Thus, a new paradigm is needed that involves integrated approaches and sustainably intensifies agricultural production to close the yield gap and to achieve equitable food security (see Figure 1).

The present review urges the need for incorporating physiological breeding strategies into food legumes improvement pipelines; addresses the gap between breeders and physiologists; focuses on physiological traits that are to be targeted; yield gains in food legumes; the impact of abiotic stress and the importance of roots and root phenotyping in food legumes breeding programs. It also discusses the introduction of physiological traits into molecular breeding programs and the potentials of genome engineering of those traits for next-generation food legumes’ breeding.

## 2. Addressing the Breeder-Physiologist Gap

Plant breeding in the past has been dependent on generating variations by multiple crosses and screening progenies to identify cultivars with higher yield potentials. The role of a physiologist in a breeding program has always been on the pre-breeding aspects, suggesting specific physiological parameters that could be used as a screening tool. It has been more than 20 years since the gap between breeders and physiologists was identified towards the physiological understanding in breeding programs [20]. This gap is still to be bridged, at least with respect to food legume breeding programs. From a breeder’s perspective, a physiological parameter needs to be heritable, stable across multiple environments and at the same time correlate with key economic traits that are of their specific interests for them to be incorporated into their programs. However, most of the physiological traits are highly variable and require high throughput screening methodologies to screen them. The reductionist approach followed by physiologists and geneticists in order to dissect specific traits identifies key genes associated with the traits, which can then be used by plant breeders [21]. To exploit the advantages of physiology in breeding pipelines, the number of traits and plots analyzed need to be increased and phenomics can greatly help achieve this. In the case of drought breeding, physiologists will have to design experiments to select drought adaptive cultivars based on the mechanism in question. For example, is the experiment measuring soil water deficit or stress avoidance? [22]. In studying complex traits such as yield and drought tolerance, only an understanding of intra- and inter plant interactions in varying environments will aid us to link the genes and those specific traits [23]. Nevertheless, it is important for breeders to work hand-in-hand with physiologists to understand the complex interactions between traits and bridging the gap that exists with physiologists can prove beneficial to their breeding projects.

One of the main challenges of integrating plant physiological studies with breeding programs lies in the laborious and time-sensitive measurement of certain physiological traits. For this reason, a limited number of genotypes are often used in physiological studies. We would like to further elaborate on this from our personal experience. The association mapping study for identifying genomic regions associated with in vitro pollen germination under heat stress in field pea (*Pisum sativum*) failed to yield any significant single nucleotide polymorphism (SNP) markers [24]. Phenotyping of this trait is both technically challenging and time-sensitive. It took approximately 5 h to run the in vitro pollen germination test on one replication of mapping individuals with two temperature treatments (control versus high temperature). Pollen viability and vigor may be reduced between the first few varieties compared to the last few varieties [25]. The results may also be confounded by the time of sampling and developmental stages. Flower samples from several plots were not sampled at the optimal flower bud stage. The optimal stage for flower sampling was at Stage III—after anther dehiscence and before flowers were fully open [25], but in the study, some samples might have been collected from a later stage, because once the flowers were fully open and the standard petal closed, they would appear similar to Stage III. Pollen viability from the later stage was lower compared to Stage III under control conditions [25]. The above problems could have been alleviated with a repeated check cultivar in the field. In this way, researchers could have run the in vitro pollen germination assay on the check cultivar after every ten samples, so they could compare the repeated check to standardize and calibrate their data to improve the accuracy of the phenotyping. Therefore, attention is required to improve experimental design and careful flower sampling (anthers at the exact and same stage) for the successful mapping of pollen vigor for an association mapping panel.

Another example of laborious and time-sensitive measurements of physiological traits is measuring photosynthetic parameters using portable photosynthesis measuring units such as Li-Cor. The main challenge is that the efficiency of photosynthesis changes over time, so the measurement should be completed within a limited amount of time, which is not practical for a breeding study that usually involves hundreds of individuals. High-throughput phenotyping offers potential in this regard. New cameras, sensors, and automatic mechanical devices such as drones (unmanned airborne vehicles, UAVs) have enabled fast, accurate, and non-destructive phenotyping at the whole-plant and canopy level [18]. For example, high-throughput chlorophyll fluorescence imaging has been used as a proxy for the underlying process of photosynthesis [26]. Identification of appropriate traits and their proper phenotyping are critical in physiological breeding strategies.

## 3. Traits to Be Targeted in Physiological Breeding

One of the best examples of the vital role of physiological traits’ linkage to yield potential is the winter wheat study in Henan province, China [27]. In the study, δ^13^C was found to be associated with an increase in harvest index, kernel number per square meter and aboveground biomass. Crop physiology, combined with crop modeling and phenotyping relevant traits, can lead to a better understanding of a particular plant mechanism. To double global wheat yields, screening for the stability of traits across wider environments, canopy architecture and its function, photosynthesis efficiency, crop phenology, and source to sink relationship in partitioning the resources available is of paramount importance [28].

In the context of drought phenotyping, in addition to the above-mentioned traits, root architecture, early vigour, flowering time, carbon isotope discrimination (CID), stomatal conductance, abscisic acid (ABA), osmolytes, chlorophyll concentration, and remobilization of water-soluble carbohydrates are to be screened to draw meaningful correlations [29]. Among these, the root architecture plays an important role and is discussed exclusively elsewhere in this review. As part of metabolomics-based phenotyping of compounds that regulate physiological processes in plants, analyzing the guard cell metabolome can provide insights into stomatal movement and elucidate transpiration control in plants [30]. Stomatal density is a simple but efficient parameter to associate with transpiration and photosynthesis efficiency of plants. Some physiological traits such as nitrogen use efficiency (NUE), which is controlled by multiple genes, are challenging to phenotype and appropriate experimental designs should be employed in doing so [31]. Efforts are underway to improve photosynthesis in crop plants aiming at increasing crop yields to meet the world’s goods and bioenergy demand. Improvement in light and carbon capture and conversion in addition to designing smarter crop canopies have been identified as potential opportunities to redesign crop photosynthesis [32]. An efficiently planned and careful phenotyping, backed up by relevant experimental designs, will narrow the gap between genotype and phenotype [29]. The influence of engineering and other technologies has introduced many next-generation phenotyping platforms and facilitates non-invasive measurements of growth and developmental traits [33]. A list of traits that can be included in a physiological breeding program is shown in Table 1.

## 4. Achieving Yield Gains in Food Legumes

In discussing the yield potential to meet the target of feeding the world in 2050, [37] pointed out that the crop production growth rate should approximately be 2.4% every year from then. However, the yield increases among the major food crops, wheat, rice, maize and soybean were only about 0.9% to 1.6% per year, which was identified as a crisis. Plant breeders strive to develop advanced varieties with a high yield potential and better nutrient composition [38] through random recombination trailed by a selection for those traits [39]. In this situation, non-leguminous crop breeding programs, aiming at increasing their yield through efficient crop nutrient management practices, can benefit from biological nitrogen-fixing capabilities of legumes through intercropping. The increased demand for plant-based products and alternative sources of proteins, tapping into the potentials of food legumes and strategies to increase their yield potential can come to our rescue.

In the past, crop yield increases have been attributed to efficient biotic stress tolerance of crops, efficient agronomic practices, resistance to abiotic stresses, and superior yield potential genetics [40]. Reports indicated that a congregation of appropriate adaptation traits could confirm increased crop yields when grown in appropriate production environments [41]. The yielding ability of crops cannot be directly determined by an individual physiological or morphological mechanism [42]. A combination of characteristics, rather than a single trait, is needed for yield improvement in drought-prone environments [43]. For example, several physiological changes have complemented the improvements in soybean yield including a greater season-long canopy interception and improved resistance to lodging, better efficiencies of converting light energy into aboveground biomass, and the partitioning of biomass to seeds [44]. Diverse morphological traits and their combinations have been associated with regions of varying geographical origin, with respect to yield improvements, as in the case of some abiotic and/or biotic stress tolerance traits. Though the production area of soybean is projected to expand [45] a rise in temperature by 2050 could cause a yield stagnation [46].

The phenology of crops in earlier reproductive phases is critical in escaping environmental constraints such as heat stress. It is only during the early vegetative growth that the first initiations of reproductive organs happen [47]. The photosynthetic assimilation rate, which could directly translate to increased yield, is 50 % higher at the time for flowering and pod formation compared to the vegetative stages in chickpea [48]. Environmental factors such as temperature, photoperiod, soil moisture content, growing season temperature, and their variables on the growth and yield of food legumes under field conditions are crucial. Thus, it is necessary to study a diverse group of germplasms under high temperature production environments to understand plant adaptation. Using simple visual scores for floral abortion, and the number of pods filled per plant, genotypes can be scored as tolerant to heat stress [49]. Chickpea is one of the indeterminate annual legumes that flowers and sets pods simultaneously [50]. Knowledge of the genetic and physiological basis of the interaction between flowering time genes and the environmental factors such as temperature and photoperiod are helpful in selecting varieties for particular environmental conditions. For example, in field pea, the photo-thermal unit received during the vegetative phase had a positive and significant correlation with yield. However, the unit received during the reproductive phase of the crop had a negative effect with several other plant traits [51]. The genetic potential of the plant to build its biomass in the vegetative phase favors the production of its threshold number of reproductive units (nodes and branches) under the influence of the environmental factors [52]. Grain yield in major crops, such as lupin, is significantly correlated with the duration of first to last flowering, last flowering to physiological maturity, and first flower to physiological maturity [53].

In terms of freezing tolerance, only a limited level of tolerance has been reported in food legumes. The lack of information on the genetics for tolerance to chilling in a range of temperatures at multiple growth stages such as germination, flowering, and pod setting is evident. In the process to develop new frost-tolerant legume germplasms suitable for Australian farming conditions, field trials confirmed that increasing the duration of the cropping cycle had a beneficial effect on plant productivity if the crops managed to survive colder weather conditions [52].

A decrease in soil moisture content can negatively affect early plant growth and development traits such as branch number, leaf area, aboveground biomass, and plant height. A positive correlation between an increased seed yield and water use in legumes has been reported in chickpea and lentil [42]. Chickpea seeds emergence and early growth is affected by the soil water content at sowing [54]. Water deficits imposed during flower development of chickpea varieties also affects the subsequent capacity of pollen germination [50]. The temperature and photoperiod thus act sequentially and most crops flower earlier on warmer days and when the photoperiod was longer than a certain minimum [53]. This is because the longer the photoperiod at any temperature, the faster the thermal sum required for flowering is accumulated [55]. Photoperiod influences and regulates the total dates of early vegetative growth and floral bud growth [56]. Reports indicate that in chickpea, photoperiod sensitivity commenced on different days after emergence in different accessions [57]. Exploitation of early flowering genes from cultivated germplasm can be beneficial in breeding photoperiod efficient pulse crops [58].

The gene clusters responsible for floral, leaf and root development, the nodulation process, and immature and mature seed development were identified to govern organogenesis at specific developmental stages of the plant [59]. Strategies that address the growth parameters under the variable field conditions will enable the capture of biomass into yield components such as seed number and seed size [60]. Double podding, early flowering, and bush habit parameters are to be considered in developing stable chickpea varieties under the Mediterranean conditions [61].

A continued supply of genetic diversity, including new or improved variability for target traits, is key for successful yield improvement in crops [62]. Photosynthesis and the associated resource-use efficiency traits offer excellent opportunities to increase yield potentials. Increasing photosynthetic capacity by studying the catalytic properties of Rubisco, canopy level photosynthesis, and maximizing its utilization by partitioning to vegetative and reproductive structures efficiently can generate higher yields in crops [63]. In deciphering yield potential mechanisms, it is important to note that individual yield attributes are often negatively correlated with each other. Only with a thorough physiological understanding of these yield attributes and their negative relationship will guide us towards manipulating them either through conventional or gene editing assisted breeding strategies [64].

## 5. Abiotic Stress Tolerance in Food Legumes

Drought and temperature stress (heat and cold) are the most common abiotic stresses affecting yield production in food legumes [65]. The negative effects of drought and temperature stress vary, depending on timing, duration, and intensity [66,67]. Other abiotic stresses that are region specific include salinity, flooding, nutrient deficiencies and toxicities, and soil acidity and alkalinity [65]. Abiotic stresses primarily cause effects such as decreased water potential and cellular dehydration that directly modify cellular biochemical properties, leading to secondary effects. The secondary effects include alternation of metabolic activity, ionic toxicity, accumulation of reactive oxygen species (ROS), and the loss of cellular integrity, which may result in cell death [68]. In the field, crops are often exposed to multiple abiotic stresses simultaneously.

Drought, salinity, and frost have overlapping mechanisms in plants. The primary responses of these three stresses are reduced water potential and cellular dehydration (osmotic stress). Osmotic effects induce the accumulation of abscisic acid (ABA) that regulates adaptive responses under conditions of drought and salinity in plants [69]. Besides that, salinity also causes ionic toxicity in plants [68]. Drought decreases the rate of symbiotic nitrogen fixation (SNF) in legumes, due to: (1) The accumulation of ureides in nodules and shoot [70]; (2) a reduction of metabolic enzyme activity [71]; (3) a decline in transpiration rate and xylem translocation rate [71]. Certain legumes (i.e., faba bean, pea, and chickpea) that export amides (primarily asparagine and glutamine) in the nodule xylem are generally more tolerant to water deficits, compared to other legumes (i.e., soybean, cowpea, and pigeon pea) that export ureides (allantoin and allantoic acid) [72]. Oxidative damage induced by drought stress negatively affects nodulation [73]. Nodules with increased enzymatic antioxidant defenses are more tolerant to drought and salinity stress in the common bean [74].

Both heat and cold stresses modify the fluidity of phospholipid cell membranes [75]. Cool season food legumes are generally cold tolerant and heat susceptible, whereas warm season food legumes are cold susceptible and heat tolerant [65]. Reproductive development in annual crop species is considered to be among the most sensitive phase of the life cycle in response to environmental stresses [76]. Heat stress immediately before or during anthesis induces sterility in many plant species, especially for pulse legumes [77]. High temperatures during reproduction reduce pollination and cause abscission of floral buds, flowers and pods; all of which result in a substantial yield loss in field pea, common bean, cowpea, and chickpea [78,79,80,81].

Heat stress causes the accumulation of heat shock proteins (HSPs) that act as molecular chaperones to correct protein misfolding and prevent protein denaturation [82]. Freezing stress induces symplastic ice crystal formation. Flooding stress reduces O_2_ availability to plant cells, which hinders aerobic respiration [83]. Reactive oxygen species, including the superoxide anion, hydrogen peroxide, the hydroxyl radical, and singlet oxygen are primarily accumulated in the chloroplast [84]. ROS synthesis is exacerbated under various environmental stresses, such as high light, heat, cold, salinity, flooding, trace element toxicity, and drought [84].

Identifying stress-tolerant traits is critical to develop stress-tolerant cultivars through plant breeding [85]. Adaptive mechanisms of plants responding to environmental stress include escape, resistance by avoidance, and resistance by tolerance [86]. The adaptive trait responses to various abiotic stresses are listed in Table 2. The most effective strategy is to escape stress. Early flowering and/or early maturing cultivars may escape the heat and/or drought conditions. Management and breeding approaches, such as shifting planting date and genetic manipulation of crop phenology, have been used to facilitate plants to escape from heat stress [87]. To resist the stress by avoidance is the second-best strategy, whereas to resist stress by tolerance usually results in a yield loss [86]. Stress avoidance is the ability of plants to avoid stress through physiological mechanisms. Stress tolerance is the ability of plants to survive under stress and they usually produce a lower economic yield compared to under optimal conditions [88]. The terms heat tolerance and heat resistance are also used in the literature interchangeably to mean a trait with stress robustness compared to heat susceptibility.

Drought adaptive traits include higher water use efficiency (WUE), a deep root system, accumulation of osmolytes and antioxidants, and synthesis of cuticular wax [85]. A deep root system improves water and nutrient extraction in the soil profile. A thicker cuticle decreases transpiration. Pea varieties with thicker or a more epicuticular wax load have lower canopy temperatures suggesting that incident radiation is more efficiently reflected by the increased amount of wax alleviates the damage of water loss and its associated heat stress [89]. Pea varieties with a more pallid green leaf color (possibly an indicator of lower levels of chlorophyll and antenna complexes at the photosystem II reaction center) absorbed less radiation [89]. Under abiotic stresses such as salinity, water, and heat, various plant species may produce different osmolytes including sugars, sugar alcohols (polyols), proline, tertiary and quaternary ammonium compounds, tertiary sulphonium compounds, γ-4-aminobutyric acid, and glycinebetaine, which in turn improve stress tolerance [68]. Furthermore, evaporative cooling capacity allows plants with access to sufficient water to maintain leaf temperatures less than 45 °C, even under hot conditions. However, poor air circulation within the canopy reduces the rate of leaf evaporative cooling [68].

Plants with indeterminate growth are generally more stress tolerant compared to the ones with a determinate growth habit. For example, heat tolerance of field pea can be improved by growing cultivars with indeterminate habits and a long duration of flowering. Thus flowering lasts for a long period during the growing season [90]. The manipulation of flowering time regulation is of considerable importance to allow certain genotypes to avoid the most stressful phases during the growing season. Photoperiod, temperature (vernalization and post-vernalization), and genotype are crucial factors for the time of flowering in pea [91]. Flowering at different nodes is not synchronous due to the indeterminate type of growth habit in pea. The field pea flowers in a sequential manner and in turn the flowering duration vary greatly because of the high degree of variability in the number of reproductive nodes [92] and late formed branches, which flower later during the reproductive phase. One of the main restrictions in screening accessions for abiotic stress tolerance in the field is the technical complexity involved in phenotyping the traits. Traits screened under greenhouse conditions may not translate well when the plants are subsequently subjected to field trials and screening. In these cases, screening crops in the greenhouses with a large-container phenotyping system to mimic field conditions and imposing abiotic stresses to select efficient genotypes could be successfully employed for pre-breeding selections [93].

## 6. Root and Root Phenotyping in Food Legumes

Research on food legumes has focused on above ground agro-morphological traits given the ease of measurements and manipulations [110]. Studies on below ground structures such as roots and nodulation have been limited by a combination of factors. Firstly, the lack of funding geared at root studies has made work in this area limited. Secondly, studying roots in different environments, coupled with the task of soil excavation and root washing, makes their assessment a daunting process. Also, the plasticity of roots grown under different conditions makes their study challenging. By devising efficient protocols to screen roots and root system architectures (RSA) of food legumes, those hidden parts could be explored for newer strategies to address global food security. Some studies have focused on either root biomass alone or in combination with root length [111]. While this gives some insight into the plant’s response under a given abiotic stress, it fails to provide a comprehensive understanding. Furthermore, it has been shown in rice [112] that investigating only the root biomass can be misleading as some genotypes tend to invest more in root length rather than biomass. This behavior has also been observed in the wild lentil, *Lens ervoides* L-01-827A [113]. Another challenging issue with analyzing root traits in food legumes, such as chickpeas and lentils, is the variation in the length of time to maturity observed among cultivars and between wild, cultivated and recombinant inbred lines (RILS); making it very difficult to identify an ideal time to assess roots during plant growth [114]. Only recently different plant cultivation systems, growth media as well as root imaging tools [115] have been developed to study root traits. Again, the physiologist-breeder collaboration should set out goals geared at achieving both developmental and breeding objectives given the plethora of different phenotyping systems available for root trait analysis. Breeders would be more inclined to accept any phenotyping systems that have been proven true in the field. However, how phenotyping systems and imaging tools can be adapted to assess legume roots has not been demonstrated on a larger scale, except RhizoTubes was shown to be effective in evaluating root architecture under water-stressed conditions in pea and other crops [116]. An outstanding question is how the huge amount of data generated would be interpreted and transferred to breeders in a coherent manner.

The contribution of legumes to soil carbon and nitrogen fixation in cropping systems has been widely reported to be beneficial to subsequent crops such as wheat [117]. Some research has also been carried out on the root architecture of lentils, chickpeas and field pea [118] grown in rotation with cereals in the Canadian prairies. However, these studies focused on the importance of food legumes in rotations but did not identify the different root traits that would give food legumes an advantage during adverse climatic conditions such as drought. Another aspect of root architecture that needs to be exploited is root diameter classes in food legumes. The correlation between root size and its function has been shown in other crops [119]. The classification of legume roots into functional diameter classes is another avenue where breeders and physiologists could collaborate. This will help identify root traits and quantitative trait loci (QTLs) that can enable food legumes to strive under adverse conditions [120]. An extensive field study demonstrated that root traits such as root length, surface area, root volume, and the number of root tips varied across different diameter classes between pulses and in some cases, were significantly higher than those of wheat and oilseed crops [118,121]. Legumes have also been shown to extract less water from deeper soil layers compared to wheat in Canadian prairies [122]. The study in Reference [123] compared the root distributions in field pea, chickpea, and soybean and found that both field pea and chickpea allocated roots into deeper soil layers unlike, soybean. However, these studies lacked further assessments on the functional implications of the differences in root traits categorized in different diameter classes and at different soil depths.

Another avenue below ground that needs to be exploited to its maximum potential is nitrogen fixation. A wealth of literature on nitrogen fixation exists. Yet farmers continue to apply nitrogenous fertilizers in their fields, with the resulting gases compounding the effects of climate change. There are also a wide range of associations between rhizobia species and legume roots; especially their wild relatives [124]. How much nitrogen is fixed depends on the legume cultivar and the environment under which it is grown [125]. Altogether, understanding the hidden half of food legumes can lead us to modify its architecture, breed crops that efficiently use nutrients and thereby generate high yielding and climate resilient crops.

## 7. Molecular Breeding of Physiological Traits

Engineering and other technological advances have transformed agricultural research into a whole new level and owing to this, designing “smart” crops is now possible. Next-generation sequencing technologies have revolutionized crop improvement programs and have enabled us to understand the nature of complex traits. In the past, except for soybean, all most all other legumes have had meager attention when it comes to genomic information and were termed as “orphans” in the genomic era [126]. However, just within in the last decade with constant and collaborative efforts from different international organizations, we now have genomic resources available for a number of grain legumes including but not limited to soybean, chickpea, pigeon pea, common bean, adzuki bean and groundnut [127]. These genome sequences, along with other omics related studies such as metabolomics, proteomics, and ionomics will aid us in the functional characterization of certain traits and expedite crop improvement. With the assistance of this genomic information, breeders have already mapped drought tolerance and disease resistance in chickpea, rust resistance and oil quality in groundnut and screened hybrid purity in pigeonpea, just to highlight a few examples [128].

Facilitating marker-assisted selection (MAS), the genomics resources have also contributed to the development and improvement of newer techniques such as genome-wide association studies (GWAS), genotype by sequencing (GBS), genomic selection (GS), and genome editing in plants and have advanced plant breeding to the next generation. They aid in gene discovery, complex pathway analysis and provide specific selection strategies; existing and future crop breeding programs can benefit the most from them by adopting these technologies [129]. Though marker-based strategies have tremendous potential in expediting breeding, the effect of the environment on genotypes and their interactions (G × E) can make them unsuccessful as crops or undergo significant changes in gene expression patterns during various morpho-physiological and stress-related changes [130]. Insufficient diversity in the primary gene pool of the accessions screened can also lead to failed MAS inbreeding [131]. These phenomena will be more evident when it comes to physiological traits, as they are influenced by changes in the environment and gene expression, which makes the incorporation of these traits into molecular breeding programs a little complex.

Many of the molecular breeding approaches, including MAS, that are applied to food legumes are based on prior success stories in cereal crops. A description of the use and success of MAS in pulse breeding is provided in Reference [131]. MAS is particularly successful only when the heritability of the trait of interest is high, which is not the case generally in physiological traits as they are influenced by multiple factors. Among the large number of QTLs (quantitative trait loci) reported in multiple crops, only a very few have been cloned and their functions studied. Reverse genetics offers help in identifying the candidate genes associated with specific traits and assists QTL cloning in unlocking the reasons behind adaptive evolution traits, such as the physiological ones [132]. QTL mapping of physiological traits may result in landing coincidentally on morphological and phenological trait QTLs as these traits are heavily controlled by physiological mechanisms. A list of physiological traits and/or parameters that can be mapped using QTL mapping techniques in crops is listed in Reference [133]. The photosynthetic assimilation rate, chlorophyll content, stomatal resistance, and transpiration rate to map photosynthesis, root mass, root depth, root axis length, and lateral root branching to correlate to nutrient absorption and assimilation, root nodule number, shoot mass, symbiotic nitrogen fixation (*Sym*) genes and various other regulatory genes for nitrogen fixation, reduction in chlorophyll content, number of late-coloring leaves per panicle determining senescence, photoperiod and flowering-related locus for mapping flowering, Na^+^ and K^+^ concentration for salt tolerance, carbon isotope ratio, abscisic acid concentration, CID, water-soluble carbohydrate concentration, osmotic potential for drought tolerance, photosynthetic parameters and leaf pigment composition for cold tolerance can all used to identify QTLs associated with their respective traits.

The wealthy genomic resources available for food legumes obtained by treating them as model crops, will facilitate molecular breeding of these crops and generate climate resilient, nutritionally superior crops. Capitalizing on the huge investments made in sequencing these crops, their hybrid vigor should be studied as it was the basis of yield increases in maize in the past [134]. Finally, molecular breeding strategies, rather than just complementing traditional breeding, should be incorporated as an integral part of it; providing vital genes, metabolites, markers, and screening tools to drive knowledge-based crop improvement [135,136].

## 8. Gene Editing Key Physiological Traits

Conventional breeding approaches, which include the crossing of plants with trait variations and utilization of mutagens (EMS, UV) have been recurrently used in plant breeding. The need for superior germplasm with variations in traits to develop elite cultivars to combat biotic and abiotic stresses is a major limitation in conventional plant breeding [137,138]. The new gene editing technology CRISPR (clustered regularly interspaced short palindromic repeats)/Cas9 (CRISPR associated protein 9) provides great promise for crop improvement employing precise editing of nucleotides although the acceptability of the technology is still under consideration [137]. This technology combats the limitations of conventional breeding by accelerating germplasm development with creating heritable and desired mutations in the genome without undesirable background mutations as observed in traditional mutagenesis in crop plants. Furthermore, the generation of transgene-free plants through segregation of traits in the F1 or F2 generations with intact heritable mutations can generate plants identical to those obtained by conventional breeding with a reduced cost and time [137]. The CRISPR/Cas9 nuclease directed by the guide RNA creates double-stranded breaks in the DNA, which are subsequently repaired by DNA repair mechanisms: Error-prone non-homologous end-joining (NHEJ) or homology-directed repair (HDR). The NHEJ repair mechanism is error-prone and can lead to insertion or deletion mutations upstream of the PAM site thereby altering the coding sequence. In comparison, the HDR mechanism generates specific point mutations or can be used to insert nucleotides as provided in the donor specified DNA template [139].

The CRISPR/Cas9 technology has been continuously optimized for improved genomic editing since the first report and the abilities of this technology have been constantly expanded, which include guide RNA engineering for use with multiple guide gRNAs [140], targeted base editing [141] and more recently epigenome editing [142]. The most widely used and characterized nuclease is Cas9 (CRISPR associated nuclease 9) however, utilization of cpf1 has also been recently reported [137]. Although utilization of CRISPR/Cas9 in legumes is still in its infancy, important reports from other crops showing the efficient utilization of CRISPR/Cas9 have been explained in this chapter. The examples provided below provide evidence for modifying physiological traits using CRISPR/Cas9 in other crops, which can be used to edit traits in legume crops.

This genome editing platform can be efficiently utilized for inducing both quantitative and qualitative traits in crops [143]. Creating quantitative variation using CRISPR/Cas9 has already been reported [144] by modifying cis-regulatory alleles of the genes involved in fruit size, inflorescence architecture and plant growth habits in tomato. Loss-of-function mutants targeting a 2 kb region of *SICLV3* showed strong to weak phenotypic effects in fruit size. The crossing of these mutant lines with wild-type plants resulted in a span of quantitative variation for fruit size. These authors further used the same approach to target promoter regions of compound inflorescence (*S*) and self-pruning (*SP*), which resulted in mutant plants showing quantitative variation for inflorescence architecture and plant growth habits in tomato [144]. This strategy to target cis-regulatory alleles could be used in crops for specific alleles, which may provide us a with continuum of phenotypic effects as observed in tomato. Natural QTL variants have limited variations for agronomic traits for breeding and generation of QTL variants using this approach might result in a better range of phenotypes [145]. However, the screening of these resultant mutant plants may be cumbersome and this can be overcome by using newly available high throughput phenotypic tools for screening crops for expected traits [143].

CRISPR/Cas9 can be efficiently used to target or modify qualitative traits where a desired loss-of-function outcome has been validated and previously studied. Mutations in four yield related genes grain number 1a (*Gn1A*), dense and erect panicle (*DEP1*), grain size (*GS3*) and ideal plan architecture (*IPA1*) were induced in the rice cultivar Zhonghua 11 [146]. Mutant lines edited with CRISPR/Cas9 (T2) for *Gn1A* resulted in an increased number of flowers per panicle (184–199) in comparison to wild-type plants (104 flowers per panicle). These mutant plants also showed an increased plant height and panicle length in comparison to wild-type plants. Mutant plants for *DEP1* had shorter panicles with significantly more flowers per panicle than wildtype plants. A significant increase in grain length and grain weight was observed in T2 *GS3* mutant plants. For *IPA* mutant plants, the phenotypic difference was based on the type of mutations generated and plants showing 12 or 21 bp deletions with no frame shift mutations with the activity of the protein still intact, showed a lower number of tillers with an increase in plant height, flower number and panicle length when compared to wild-type plants [146].

Drought tolerance was significantly increased by the overexpression of *ARGOS8* in maize plants using CRISPR/Cas9 [147]. The expression of *ARGOS8* in wild-type inbred lines was reported to be around 3–25 transcripts per ten million in all tissues and 260 transcripts per million in kernels whereas *GOS2* expression was reported to be 6000 transcripts per million. Hence, CRISPR/Cas9 was used to insert the maize *GOS2* promoter upstream of the 5’UTR of *ARGOS8* to increase the expression of *ARGOS8* in plant tissues. The insertion of the *GOS2* promoter was achieved using the HDR repair mechanism where the *GOS2* promoter sequence was provided as the donor template for insertion. The resultant transgenic F0 plants were crossed with wild-type inbred plants and the transgenic plants without the CRISPR components were tested in field conditions at eight locations. The transgenic plants showed a higher yield of ~5 bushels per acre more compared to the control plants in locations with flowering stress conditions and no differences in yield were obtained in locations with optimal field conditions [147]. As observed in this study, CRISPR/Cas9 technology can be used to effectively alleviate heat and drought stress by the overexpression of genes involved in heat and drought stress in crops. A detailed list of genes involved in heat and drought stress for wheat and maize has been reviewed in Reference [148]. The functional information available for agronomically important genes in other crops can be translated to food legumes by identifying orthologues of these genes in legumes—a boon in the sequencing era where genome sequences of key food legumes are available and few sequencing projects are underway [149]. The sequence information available for legumes will allow us to design specific gRNAs without any putative binding sites homologous to the gRNA in the genome to minimize off-target binding. Li and colleagues [146] analyzed off-target mutation events in rice and concluded that a minimum of a two nucleotide difference was necessary to reduce off-target binding of guide RNAs.

One of the main bottlenecks for using CRISPR/Cas9 in plants is the time required for the development of transgenic plants. Although transgene delivery can be easily performed in model plants like *Arabidopsis* using the floral dip method, challenges exist for developing stable transgenic plants in legumes. In studies involving symbiotic nitrogen fixations, the hairy root transformation method, mediated by *Rhizobium rhizogenes* in addition to stable transformation by *Agrobacterium tumefaciens*, has been effectively used in *Lotus japonicus* [150] and *Medicago truncatula* [151]. Hairy root transformation is a transient method where editing events can only be observed in the root nodules where the function of genes involved in symbiotic nitrogen fixation can be studied with almost half the time (1–2 months) required for developing stable transgenics (~4 months) [152]. With improvements being made in CRISPR/tools and in the development of transgenic plants, genome editing using CRISPR Cas9 could revolutionize the breeding of food legumes.

## 9. Conclusions

In summary, devising proper strategies to ensure food security in a world threatened by a rapid increase in population and climate change is of tremendous importance. Crop improvement programs, rather than just relying on major staple food crops, should attempt to unlock the potentials of underutilized or orphan crops. Traditionally, food legumes play a vital role in the dietary habits and livelihoods of people living in the developing countries of the world. Provided with proper research attention and framework, these crops have the capacity to alleviate hidden hunger and poverty in those nations. The biological nitrogen fixation capacity of legumes adds additional benefits to cropping systems as they save fertilizer costs. Though still in its infancy, with the newer high throughput phenotyping platforms and a vast amount of genomic resources that are now available for legumes, their breeding can be accelerated to increase genetic gains. Like the efforts made in cereal crops, food legumes should inevitably be added to next-generation breeding efforts to harness their potentials. Research projects aimed at increasing and understanding the genetic diversity of food legumes should be encouraged. The key genes that are found to be associated with physiological traits in legumes can then be incorporated into gene editing platforms to design efficient food legumes. The global pulse breeding community can build up success stories from cereal crop improvements and leverage physiological breeding. Finally, a better breeder-physiologist understanding, along with available genomics, transcriptomics, metabolomics and phenomics resources, next-generation food legumes that are nutritionally superior, stress tolerant, high yielding and environmentally sustainable can be bred.

## Figures and Tables

**Table 1:** Traits and parameters that are to be targeted in physiological breeding programs. Both non-invasive and invasive types of measurements are listed *.

Type of Measurement	Traits/Parameters	Equipment/Mode of Measurement
Non-invasive	Aboveground biomass, root architecture, seedling vigour, canopy structure and growth dynamics, normalized difference vegetative index (NDVI)	2D and 3D Imaging using stereo camera systems and laser scanning instruments, NDVI GreenSeeker
	Photosynthetic parameters, quantum yield, non-photochemical quenching	Fluorescence cameras
	Surface temperature	Thermal imaging
	Water content, leaf and canopy water status and pigment composition	Near-infrared cameras, thermal and hyperspectral cameras
	Nutrient status, pigment degradation, photosynthetic efficiency, water content, senescence and pollen viability	RGB and multispectral cameras
	Water stress, fruit maturity	Long-wave infrared and thermal cameras
	Photosystem II activity/Chlorophyll fluorescence	Pulse amplitude modulated (PAM) fluorescence, hyper-spectral spectroradiometers
	Stomatal conductance	Handheld porometer and Li-COR
	Chlorophyll content	Portable optical soil plant analysis development (SPAD) meters
	Leaf area index (LAI)	Conventional planimeter and leaf area meter
Invasive	Enzyme activity, hormone and metabolites estimation, carbon isotope discrimination	Liquid chromatography, mass spectrometry and calorimetry
Invasive and Non-invasive	Root system architecture (RSA), root biomass	Shovelomics, positron emission tomography (PET), magnetic resonance imaging (MRI) and radio detection and ranging (RADAR)

* data provided in the table are adapted from References [17,18,33,34,35,36].

**Table 2:** Adaptive traits related to abiotic stresses in legume crops.

Stress	Trait	Crop	References
Drought	High water use efficiency	Alfalfa, faba bean	[94,95]
	Vigorous root growth	Alfalfa, chickpea	[96,97]
	Osmolyte accumulation/osmotic adjustment/turgor maintenance	Alfalfa, faba bean	[96,98]
	Accumulation of antioxidants	Alfalfa	[96]
	Increased leaf cuticular wax	Alfalfa	[99]
	Early flowering/maturity	Chickpea, common bean, cowpea, faba bean, lentil	[86,100]
	Low leaf conductance/stomatal regulation/ transpiration	Faba bean	[95,96,98]
	Delayed leaf senescence	Alfalfa	[96]
	Changed leaf orientation	Soybean	[68]
	Reduced canopy temperature	Chickpea, Faba bean, soybean	[100,101,102]
	Relative water content	Faba bean	[101]
Salinity	Accumulation of osmolytes/osmotic adjustment/turgor maintenance	Field pea	[103]
Flooding	High stomatal conductance	Lentil	[104]
	Large air-spaces and aerenchyma in roots	Lentil, field pea, soybean	[104,105]
Heat	Increased pollen germination under stress	Field pea	[106]
	Reduced canopy temperature	Chickpea	[86,100]
	Accumulation of leaf cuticular wax	Field pea	[89]
	Early flowering/maturity	Lentil, field pea, chickpea	[10,86,90]
	Indeterminate growth habit	Field pea, chickpea	[25,100]
Cold	Presence of dehydrin protein	Cowpea	[107]
	Increased osmoprotectants	Faba bean	[108]
	Increased fatty acid desaturation of membrane lipids	Faba bean	[108]
	Maintenance of photosynthesis	Field pea	[109]
